# Pandemic SHV-106-producing *Klebsiella pneumoniae* ST231 isolated from Brazilian hedgehog (*Coendou spinosus*) reveals an emerging environmental circulation of a high-risk multidrug-resistant lineage

**DOI:** 10.1007/s10482-026-02317-7

**Published:** 2026-04-28

**Authors:** Jacqueline Meyer, Jéssica A. Martins, Amanda Haisi, João P. Araújo Júnior, Gustavo H. Z. Winter, Raquel F. S. Raimondo, Marcelo M. Alievi, Marcos Bryan Heinemann, Natália C. Gaeta

**Affiliations:** 1 Hospital de Clínicas Veterinárias, Universidade Federal do Rio Grande do Sul, Porto Alegre, Brazil; 2 Núcleo de Conservação e Reabilitação de Animais Silvestres, Universidade Federal do Rio Grande do Sul, Porto Alegre, Brazil; 3 Programa de Pós-Graduação Em Saúde Única, Universidade Santo Amaro, São Paulo, Brazil; 4 Universidade Estadual Paulista (UNESP), São Paulo, Brazil; 5 Faculdade de Medicina Veterinária e Zootecnia, Universidade de São Paulo, São Paulo, Brazil

**Keywords:** Hedgehog, *bla*_SHV-106_, Surveillance, One Health

## Abstract

Antimicrobial resistance (AMR) poses a major global health challenge, and wildlife has increasingly been recognized as an important sentinel for monitoring the environmental circulation of clinically relevant resistant bacteria. In this study, we report the phenotypic, genomic, and phylogenomic characterization of a multidrug-resistant (MDR) *Klebsiella pneumoniae* isolate recovered from a free-ranging wild porcupine (*Coendou spinosus*) in southern Brazil. An extended-spectrum β-lactamase (ESBL)-producing strain, designated UFRGS-ourico-23, was isolated during routine surveillance at a Wild Animals Care Unity. It exhibited resistance to β-lactams, quinolones, aminoglycosides, sulfonamides, and tetracycline. Whole-genome sequencing revealed a complex resistome, including *bla*_SHV-106_, *oqxAB*, *qnrB1*, aminoglycoside-modifying enzymes, sulfonamide, tetracycline, fosfomycin, and trimethoprim resistance genes, consistent with the MDR phenotype. Notably, ESBL production was mediated by the rare *bla*_SHV-106_ gene chromosomally located near the lactose operon. In addition, the UFRGS-ourico-23 strain belonged to the pandemic high-risk sequence type ST231. This is the first report of a *bla*_SHV-106_ in an animal-derived *K. pneumoniae* ST231 in Brazil. The strain also harbored a class 1 integron and conjugative IncFIB and Col440I plasmids carrying multiple resistance genes, as well as heavy-metal tolerance operons, suggesting co-selection pressures in anthropized environments. The phylogenomic analysis demonstrated close clustering with globally distributed, predominantly human-derived ST231 genomes, indicating limited core-genome divergence. Collectively, these findings document the emergence of a high-risk MDR *K. pneumoniae* ST231 clone in Brazilian wildlife and highlight the role of wild animals as reservoirs and sentinels of clinically relevant AMR lineages, reinforcing the need for integrated One Health genomic surveillance at the human–animal–environment interface.

**Supplementary Information:**

The online version contains supplementary material available at 10.1007/s10482-026-02317-7.

## Introduction

The global rise of antimicrobial resistance (AMR) is one of the most critical threats to public health, with alarming projections of mortality and socioeconomic impact for the coming decades (WHO 2021). Among the resistance mechanisms with the most significant clinical impact are extended-spectrum β-lactamases (ESBLs), enzymes capable of hydrolysing penicillins, third- and fourth-generation cephalosporins, and monobactams (WHO 2024). Amongst the genes coding ESBLs, the *bla*_SHV_ gene family remains one of the most significant determinants in enterobacteria, often associated with hospital outbreaks and multidrug resistance (Husna et al. 2023).

*Klebsiella pneumoniae*, an opportunistic pathogen of global relevance, is a significant reservoir of resistance genes. The emergence of high-risk clones has been documented by their exceptional capacity for international spread and association with multidrug resistance (MDR) phenotype (Peirano et al. 2020). Although historically restricted to nosocomial environments, the circulation of these clones in natural ecosystems raises serious concerns about the interface between humans, animals, and the environment, reinforcing the need for a One Health approach (Wyres and Holt 2018). In this context, wild animals have gained prominence as epidemiological sentinels of environmental antimicrobial resistance (Pérez Maldonado et al. 2025). Species that inhabit anthropomorphised regions or maintain direct contact with human effluents and waste can act as reservoirs or disseminators of MDR bacteria between different niches (Quintela et al. 2024).

The *Coendou spinosus* porcupine is a small to medium-sized rodent whose geographical distribution covers the tropical and subtropical forests of the Atlantic Forest in south-eastern Brazil, extending from the State of Espírito Santo to Rio Grande do Sul. It is also found in northern Uruguay, north-eastern Argentina and eastern Paraguay (Voss 2011). The species has a predominantly herbivorous diet based on leaves, fruits, and tree bark, with occasional consumption of flowers. Although its feeding behaviour is typically arboreal, observations document foraging on the ground (De Abreu et al. 2016). The ongoing process of habitat fragmentation has confined populations of this species to forest remnants within urban and peri-urban areas, thereby corroborating their proximity to anthropised areas and exposing the animals to sources of contamination, such as domestic effluents and hospital waste, thereby creating an environment that favours colonisation by multi-resistant human pathogens (Veríssimo et al. 2022).

Herein, we report the phenotypic and genomic features of a *K. pneumoniae* belonging to the pandemic and high-risk sequence type (ST) ST231, carrying *bla*_SHV_, isolated from a hedgehog (*Coendou spinosus*) from Brazil.

## Methods

During a surveillance of ESBL-producing Enterobacteriaceae conducted in wild animals attended in the Wild Animals Care Unity (WACU) in the Federal University of Rio Grande do Sul, Brazil, a rectal swab sample was obtained from a wild hedgehog (*E. europaeus*) and refrigerated in Stuart transport media until processing. The swab was inoculated onto MacConkey agar (Acumedia, India) supplemented with ceftriaxone (2.0 µg/mL) to detect ESBL-producing Enterobacteriaceae (Jacob et al. 2020). Plates were incubated at 35 ± 2 °C under aerobic conditions for 24 h. After plate analysis, colonies were separated, and bacterial species were determined using Matrix-Assisted Laser Desorption/Ionization Time-of-Flight (MALDI-TOF).

Antimicrobial susceptibility profile of each isolate was tested using the disk-diffusion test (Kirby-Bauer method) using the following class drugs (DME, Brazil): amoxicillin-clavulanate (10 µg), aztreonam (30 µg), cefepime (30 µg), cefotaxime (30 µg), cefoxitin (30 µg), ceftazidime (30 µg), ceftriaxone (30 µg), ertapenem (10 µg), imipenem (10 µg), meropenem (10 µg), ciprofloxacin (05 µg), gentamicin (10 µg), nalidixic acid (30 µg), sulfonamide-trimethoprim/sulfamethoxazole (1.25/23.75 µg), and tetracycline (30 µg) (CLSI 2023). Finally, ESBL production was confirmed by the double-disk synergy test with cefepime (30 µg), cefotaxime (30 µg), ceftazidime (30 µg), ceftriaxone (30 µg), and amoxicillin-clavulanate (10 µg) (Kaur et al. 2013). Multidrug resistance (MDR) was defined as resistance to at least three antimicrobial classes (Magiorakos et al. 2012).

After ESBL confirmation, the strain was sequenced. DNA was extracted using the PureLink® Genomic DNA Mini Kit (Thermo Fisher Scientific, USA) according to the manufacturer's protocol and stored at − 20 °C. DNA concentration was assessed using the Qubit™ dsDNA High Sensitivity Assay Kit (Thermo Fisher Scientific, Waltham, MA, USA). Library preparation was elaborated with approximately 350 ng of dsDNA with Illumina COVIDSeq (Illumina Inc., San Diego, USA). The library was cleaned with 0.9 × Illumina Tune Beads and eluted in 50 µL of resuspension buffer. Sequencing was performed on the MiSeq Platform (Illumina Inc., San Diego, CA) using 2 × 300 bp reads.

Reads were quality assessed, trimmed, and filtered using FastQC v.0.12.1 (https://github.com/s-andrews/FastQC), TrimGalore v.0.6.10 (https://github.com/FelixKrueger/TrimGalore), and Trimmomatic v.0.39 (https://github.com/timflutre/trimmomatic), respectively. De novo assembly was conducted with Unicycler v.0.5.1 (https://github.com/rrwick/Unicycler), and annotation was performed using Prokka v.1.14.0 (https://github.com/tseemann/prokka). The resistome was in silico predicted using CARD (https://github.com/arpcard), the Bacmet v.2.0 (http://bacmet.biomedicine.gu.se/), and ResFinder v.4.7.2 (http://genepi.food.dtu.dk/resfinder) databases. PlasmidFinder v.2.1 (https://cge.food.dtu.dk/services/PlasmidFinder/) and mlplasmids v.2.2.3 (https://gitlab.com/sirarredondo/mlplasmids) were used to predict the plasmidome. MOB-Suite v.3.1.0 (MOB-recon) was used to reconstruct plasmids from draft assemblies (https://github.com/phac-nml/mob-suite#docker-image). Virulome was assessed using the Virulence Finder Database (VFDB v.2.0.1) in the ABRicate tool v.1.0.1. The presence of integrons was determined using Intron Finder (https://github.com/gem-pasteur/Integron_Finder), and their composition was analyzed using blastp (https://blast.ncbi.nlm.nih.gov/Blast.cgi). Finally, Kleborate (https://github.com/klebgenomics/Kleborate) was used to confirm species and assess ICEKp-associated virulence loci, virulence plasmid-associated loci, and capsule (K) and LPS (O) serotype prediction.

The isolated strain was analyzed using 40 genomes with the same ST, sourced from PubMLST and BV-BRSV databases. The genomes were selected and downloaded based on epidemiological data (e.g., country and year of isolation, and host source; Supplementary Table 1) and based on quality (≤ 5% contamination, ≥ 90% completeness) using CheckM v.1.2.2 (https://github.com/Ecogenomics/CheckM/). Phylogenies were inferred using the CSI Phylogeny 1.4 tool (https://cge.food.dtu.dk/services/CSIPhylogeny/) using the default parameters, based on the concatenated alignment of single-nucleotide polymorphisms (SNPs). Phylogenetic analysis was performed with FastTree 2 using the maximum-likelihood algorithm (1,000 bootstrap replicates). Finally, the pan-resistome with clustered genomes from UFRGS-ourico-23 was analyzed using PRAP (https://github.com/syyrjx-hyc/PRAP/).

## Results and discussion

During surveillance, an ESBL-producing *K. pneumoniae* strain, named UFRGS-ourico-23, was isolated from a female wild hedgehog (*Coendou spinosus*) received at the WACU. This hedgehog was admitted to the Care Unit due to a dog bite. It was cleared for release after about a month.

The UFRGS-ourico-23 strain was whole-genome sequenced, leading to 3,484,964 reads and approximately 191 × coverage (Table 1). It displayed an MDR phenotype, with resistance to β-lactams, quinolones, aminoglycosides, sulfonamides, and tetracycline (Table 1). The genomic analysis supported these findings, identifying resistance determinants for β-lactams (*bla*_SHV-106_), quinolones (*oqxA, oqxB, qnrB1*), aminoglycosides (*aadA2, aph(6)-Id, ant(3")-Ia, aph(3")-Ib*), sulfonamides (*sul1, sul2*), tetracyclines (*tetA*), fosfomycin (*fosA*), and trimethoprim (*mph(A)*) (Table 1).

**Table 1:** Genomic data of *Klebsiella pneumoniae* ST231 (UFRGS-ouriço-2023 strain) retrieved from a wild hedgehog from Brazil

Characteristics	UFRGS-ourico-2023
Source	Rectal swab—hedgehog
Species	*K. pneumoniae*
ATB resistance profile^a^	AMC, ATM, CRO, CTX, CAZ, CPM, CIP. GEN, NAL, SUT, TET
Genome Size (bp)	5,421,792
No. of CDSs^b^	5,083
G + C content (%)	57.18
N50 (bp)	108,437
tRNAs (*n*)	64
rRNAs (*n*)	1
MLST (ST)^c^	231
Resistome	
*β-lactams*	*bla*_*SHV-106*_
*Aminoglycosides*	*aadA2, aph(6)-Id, ant(3’’)-Ia, aph(3’’)-Ib*
*Fosfomycin*	*fosA*
*Macrolides*	*mph(A)*
*Quinolones*	*oqxA, oqxB, qnrB1*
*Sulfonamides*	*sul1, sul2*
*Tetracyclines*	*tet(A)*
*Trimethoprim*	*dfrA12*
*Heavy metals*	*arsABCDHR, copAS, cueR, cusBCFR, cutA, merACDEPRT, nikABCDER, pcoABCDERS, silABCFPRS, znuABC, zraRSP*
*Desinfectants*	*qacE1*
Plasmidome	IncFIB, Col440I
Virulome	o*mpA, rfbB, entB, fimBCDHK, mrkD, iutA*
Genbank Accession#	PRJNA1394218: SAMN54318015

**Fig. 1:**
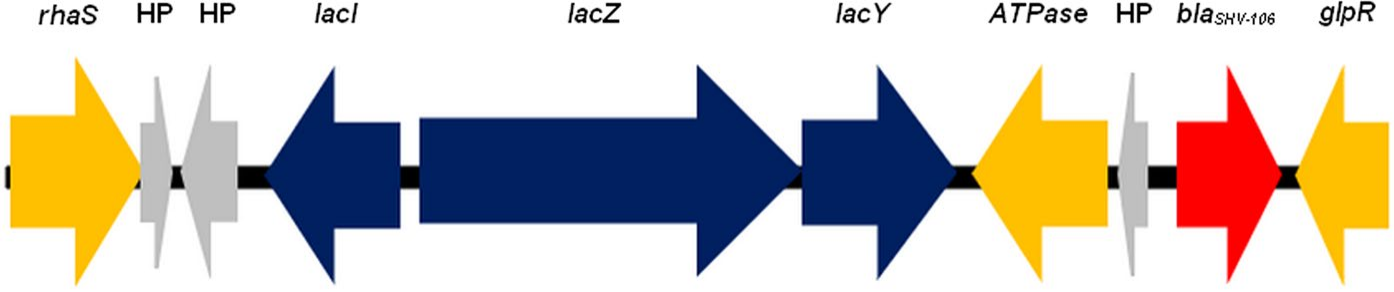
Genetic organization of the chromosomal region harboring the *bla*_SHV-106_ gene in *Klebsiella pneumoniae* ST231. The schematic representation shows the genomic context of *bla*_SHV-106_ located on the chromosome, adjacent to the lactose operon (lac). The *bla*_SHV-106_gene (red arrow) is positioned downstream of *lacY* and upstream of *glpR*. Genes involved in lactose metabolism (*lacI, lacZ*, and *lacY*) are shown in blue, while ATPase-related genes and regulatory elements are indicated in yellow. Hypothetical proteins (HP) are represented in grey. Arrows indicate gene orientation and predicted direction of transcription

ESBL production is mainly conferred by *bla*_CTX-M,_ the most abundant gene family since its acquisition by *E. coli* from the environmental *Kluyvera* spp. (Canton et al. 2012). In the UFRGS-ourico-23, however, its ESBL production is conferred by *bla*_SHV-106._ This gene is located upstream of the lactose operon (lac) on the chromosome (Fig. 1), suggesting a stable genetic configuration that may contribute to the long-term maintenance of the resistance phenotype. Similarly, a human-derived *K. pneumoniae* strain from China carried the *bla*_SHV-106_ gene on the chromosome, but downstream of the lac operon (Zhao et al. 2022). Chromosomally encoded ESBLs may reduce dependence on plasmid-mediated transfer and could represent ancestral or locally adapted resistance lineages within *K. pneumoniae* populations. It seems that *bla*_SHV-106_ is rarely reported, with cases in Portuguese hospitals (Carvalho et al. 2021) and in China (Zhao et al. 2022). To the best of our knowledge, this is the first report of a *bla*_SHV-106_-carrying *K. pneumoniae* of animal origin in Brazil.

A class 1 integron was detected in the IncFIB plasmid of UFRGS-ourico-23. It displayed a canonical gene cassette array composed of *aadA, qacEΔ1* and *dfrA12*, consistent with integron structures commonly described in *K. pneumoniae*. (Supplementary Fig. 1). Such arrangements reflect the modular nature of integrons and their capacity to accumulate resistance determinants through successive recombination events, contributing to the observed multidrug-resistant phenotype*.* Several antibiotic-resistance gene cassettes have been identified in association with class 1 integrons; however, the *aadA* gene is among the most frequently encountered (Mazel 2006). In addition, these integrons harbor a conserved segment that includes the *sul1* gene, which confers sulfonamide resistance, and the *qacEΔ1* gene, which confers resistance to quaternary ammonium compounds (Drouin et al. 2002). Other studies have shown that class 1 integrons may also harbor *mph(A)* and *drfA* genes (Xu et al. 2017), which confer macrolide and trimethoprim resistance, respectively. Finally, previous studies have reported *K. pneumoniae* isolates carrying *bla*_SHV-106_ lacking the class 1 integron (Carvalho et al. 2021; Chatzidimitriou et al. 2025).

UFRGS-ourico-23 harbors replicons of IncFIB and Col440I plasmids. IncFIB plasmids are frequently identified in *K. pneumoniae* (De Oliveira et al. 2020). Their large size and genetic plasticity characterize them, and they can carry hypervirulent carbapenem-resistant *Klebsiella pneumoniae* (Sun et al. 2025). In UFRGS-ourico-23, the IncFIB replicon was associated with multiple antimicrobial resistance genes, such as *aadA2, dfrA12* (class 1 integron)*, mph(A), sul1, sul2, aph(6)-Id*, and *aph(3’’)-Ib*, but lacked known virulence determinants, suggesting a primary role in resistance maintenance rather than pathogenicity enhancement. Finally, it was predicted as conjugative, indicating its potential contribution to horizontal gene transfer within microbial communities, notably in hospitals (de Oliveira et al. 2020) and fresh produce (Stein et al. 2024).

Furthermore, the resistome analysis of the UFRGS-ourico-23 revealed heavy-metal tolerance genes for arsenic (*arsABCDHR*)*,* copper (*copAS, cueR, cusBCFR, cutA*)*,* mercury (*merACDEPRT*)*,* nickel (*nikABCDER*)*,* silver (*silABCFPRS*)*, and* zinc (*znuABC, zraRSP*). The presence of multiple heavy-metal tolerance operons in UFRGS-ourico-23 suggests adaptation to metal-contaminated environments. Such genes may facilitate persistence in ecological niches subjected to anthropogenic pressure, where co-selection mechanisms can indirectly favor the maintenance of antimicrobial resistance genes (Baker-Austin et al. 2006).

**Fig. 2:**
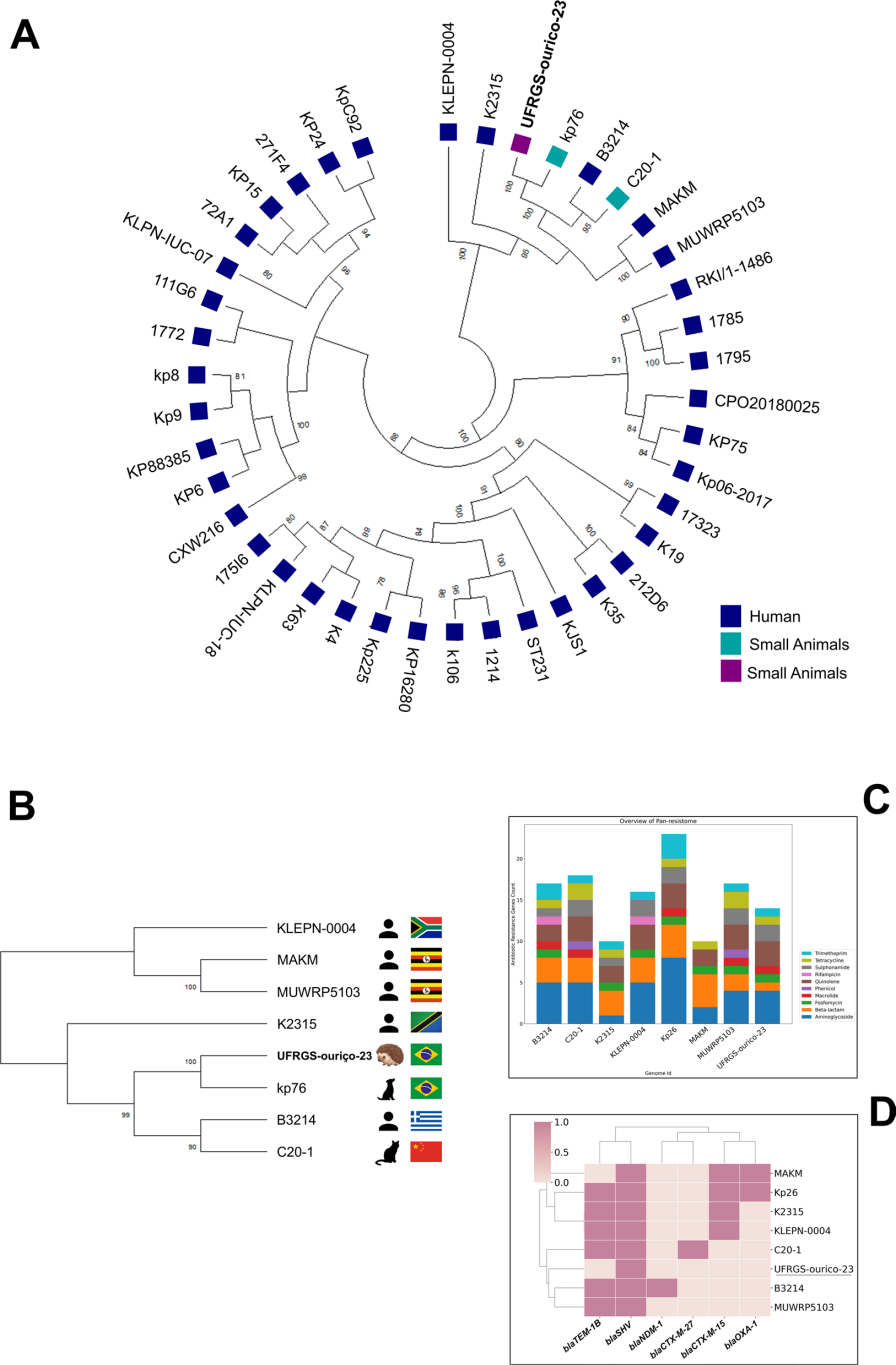
Global distribution of *K. pneumoniae* ST231. World map showing the geographic distribution of *Klebsiella pneumoniae* sequence type 231 (ST231) genomes retrieved from the BV-BRC platform. Countries are annotated with the total number of genomes available per country (N). Icons indicate the host source of the isolates, as recorded in the database metadata, including human and animal origins. This representation highlights the wide geographic distribution and host diversity of the ST231 lineage included in the comparative genomic and phylogenomic analyses

Our strain belongs to the ST231, a high-risk, worldwide distributed and successful lineage characterized by the acquisition of diverse resistance determinants (Bonnin et al. 2020). This lineage has only been reported in humans until 2018, when it was first described in a dog from Brazil (Silva et al. 2018). However, after 2018, ST231 was still reported in humans (Fig. 2). To the best of our knowledge, this is the first report of *K. pneumoniae* ST231 in a wild animal (hedgehog; *Coendou spinosus*). Finally, and interestingly, *K. pneumoniae* ST231 usually harbors *bla*_CTX-M-15_, an ESBL-related gene (Silva et al. 2018; Mancini et al. 2018). However, here we report a *K. pneumoniae* ST231 harboring the intrinsic *bla*_SHV-106_, only_._ The identification of this sequence type in a free-ranging wild hedgehog expands its known host range and ecological distribution, indicating that ST231 is not restricted to clinical settings and may circulate across different biological sources.

The virulence profile of the UFRGS-ourico-23 included genes associated with adhesion, iron acquisition, and immune evasion. The presence of fimbrial genes (*fimBCDHK* and *mrkD*) suggests a possible enhanced ability to adhere to host tissues and form biofilms (Monteiro et al. 2024), whereas *entB* and *iutA* are usually involved in siderophore-mediated iron acquisition, an essential mechanism for bacterial survival during infection (Holden et al. 2016; Paczosa and Mecsas 2016). Additionally, *ompA* and *rfbB* contribute to outer membrane integrity and lipopolysaccharide biosynthesis, respectively, which are important for immune evasion and host colonization (Monteiro et al. 2024).

**Fig. 3:**
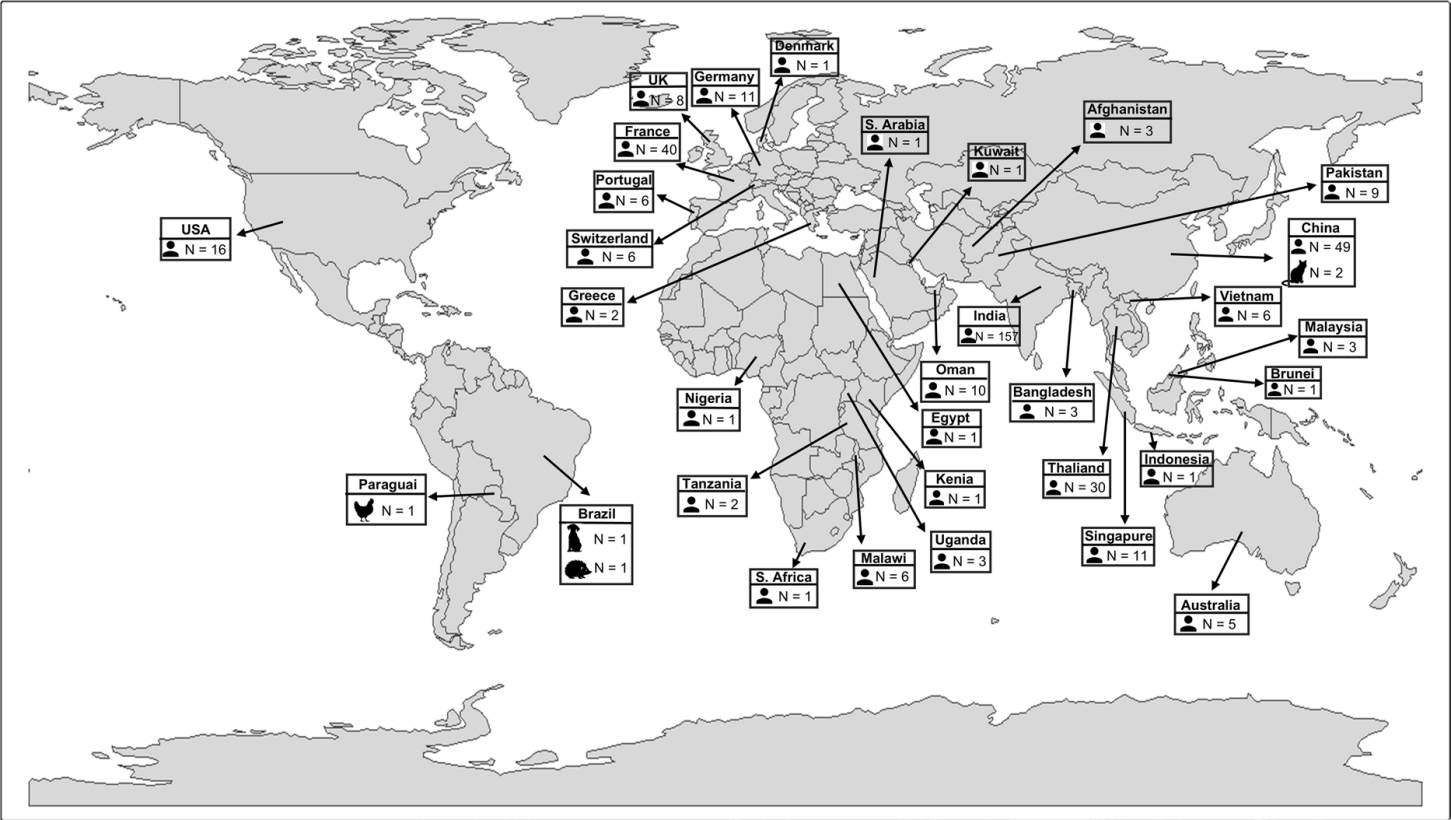
High-resolution SNP-based phylogenomic analysis of *Klebsiella pneumoniae* ST231. **A** Core-genome single-nucleotide polymorphism (SNP) phylogenetic tree illustrating the genetic relatedness among ST231 isolates. **B** Source and country of origin of ST231 genomes clustering with UFRGS-ourico-23. **C** Resistome profiles of ST231 isolates clustering with UFRGS-ourico-23 **D** Distribution of extended-spectrum β-lactamase (ESBL) and carbapenem resistance genes among ST231 genomes clustering with UFRGS-ourico-23

The high-resolution SNP-based phylogenomic analysis revealed that the *K pneumoniae* ST231 strain UFRGS-ourico-23 clustered tightly with a subset of globally distributed ST231 genomes (Supplementary Table 1), indicating a close genetic relationship and limited core-genome divergence (Fig. 3A). The limited core-genome divergence observed among ST231 genomes supports the notion of a genetically conserved lineage with a high capacity for dissemination. This clustering suggests the circulation of a highly conserved lineage across different geographic regions and sources, reinforcing the epidemiological relevance of ST231 as a successful high-risk clone. Interestingly, most isolates were human-derived, and only three animal-derived ST231 were identified in the database: from a cat (China), a dog and a hedgehog (both in Brazil). In this case, both Brazilian genomes were phylogenetically close, indicating the circulation of ST231 in pets and wild animals. The close phylogenetic relationship between the wildlife-derived isolate and human-associated strains suggests recent or ongoing exchanges between anthropogenic and natural environments. To the best of our knowledge, this is the first report of a *K. pneumoniae* ST231 in a Brazilian wild animal (Fig. 3B).

In addition, the pan-resistome analysis revealed a multidrug-resistance profile across all isolates, which clustered with UFRGS-ourico-23 (Fig. 3C). Furthermore, this analysis also demonstrated a consistent accumulation of clinically relevant antimicrobial resistance genes among phylogenetically related isolates (Supplementary Fig. 2), including determinants conferring resistance to extended-spectrum β-lactams (*bla*_SHV_) (Fig. 3D).

This study provides a detailed genomic and phenotypic characterization of a multidrug-resistant *Klebsiella pneumoniae* ST231 carrying *bla*_SHV-106_ isolated from a free-ranging wild hedgehog. The combination of chromosomal ESBL encoding, a structured class 1 integron, conjugative plasmids, and metal tolerance determinants highlights the genetic versatility of this lineage and the adaptive capacity of ST231 to acquire and maintain diverse resistance determinants. Also, ST231 has been predominantly associated with human infections and clinical settings, with only a unique report in a companion animal, highlighting a likely human-to-animal dissemination followed by environmental or interspecies dissemination. Furthermore, UFRGS-ourico-23 represents, to the best of our knowledge, the first report of the ST231 in Brazilian wildlife. Finally, antimicrobial resistance surveillance in wildlife admitted to conservation and rehabilitation centers is essential for tracking the environmental circulation and cross-sector dissemination of high-risk bacterial clones. Collectively, these findings highlight wildlife as an emerging, previously underestimated reservoir of clinically relevant high-risk clones and underscore the urgent need for integrated genomic surveillance at the human-wildlife interface within a One Health context.

## Supplementary Information

Below is the link to the electronic supplementary material.

Supplementary file1 (TIF 69 KB)

Supplementary file2 (TIF 1698 KB)

Supplementary file3 (XLSX 12 KB)

## Data Availability

The raw data is available at Genbank under the mumber PRJNA1394218: SAMN54318015. Codes are available at github.com/GaetaLab/Wild-animals.
