# Supplementary figures and images for: Pandemic SHV-106-producing Klebsiella pneumoniae ST231 isolated from Brazilian hedgehog (Coendou spinosus) reveals an emerging environmental circulation of a high-risk multidrug-resistant lineage
Source: Antonie Van Leeuwenhoek. 2026 Apr 28;119(5):105. doi: 10.1007/s10482-026-02317-7 (PMC13121183; doi:10.1007/s10482-026-02317-7)

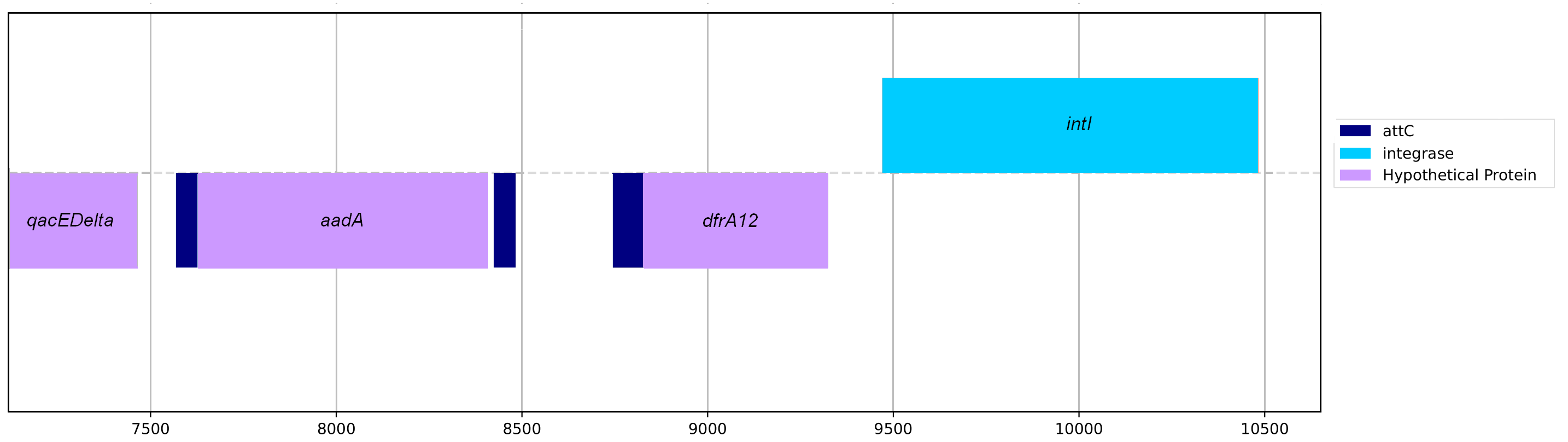

Supplement: Supplementary file 1 — Supplementary file1 (TIF 69 KB) [file 10482_2026_2317_MOESM1_ESM.tif]

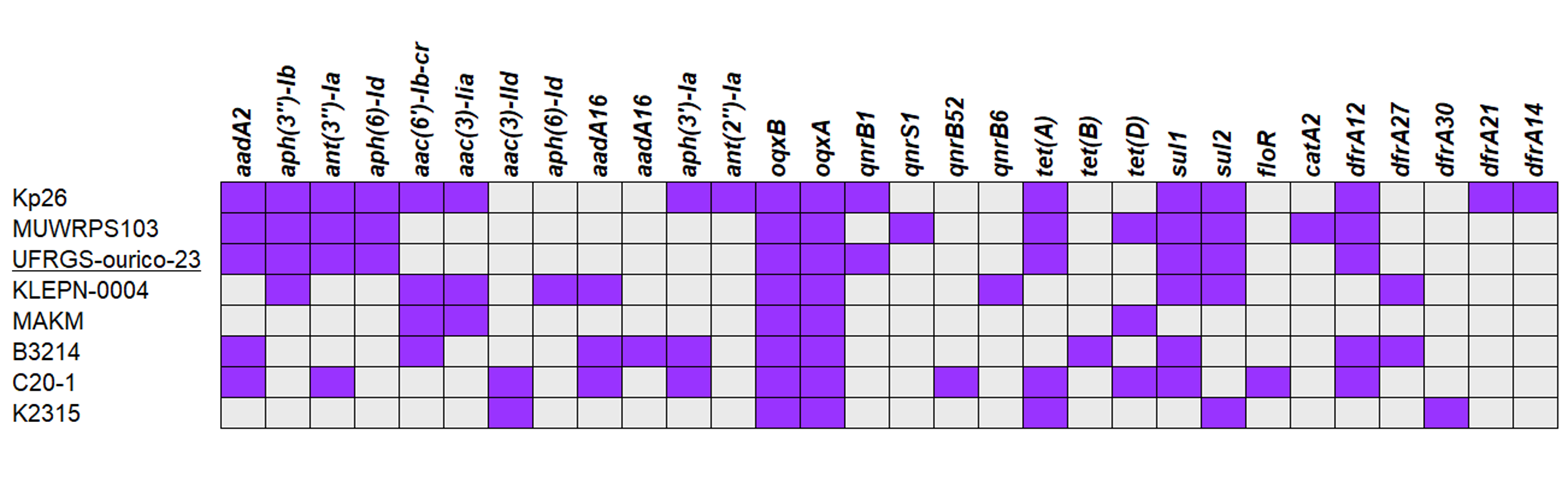

Supplement: Supplementary file 2 — Supplementary file2 (TIF 1698 KB) [file 10482_2026_2317_MOESM2_ESM.tif]
